# Adverse effects of psychotherapy: protocol for a systematic review and meta-analysis

**DOI:** 10.1186/s13643-018-0802-x

**Published:** 2018-09-08

**Authors:** Rahel Klatte, Bernhard Strauss, Christoph Flückiger, Jenny Rosendahl

**Affiliations:** 1 Institute of Psychosocial Medicine and Psychotherapy, Jena University Hospital, Stoystr. 3, 07740 Jena, Germany; 2 Department of Psychology, Division of Psychological Interventions and Psychotherapy, University of Zurich, Binzmühlestrasse 14, Box 04, CH-8050 Zürich, Switzerland

**Keywords:** Psychotherapy, Harms, Adverse effects, Adverse events, Safety, Systematic review

## Abstract

**Background:**

While it is well known that psychotherapy is efficacious in the treatment of mental disorders, much less is known about the adverse effects of psychotherapeutic interventions. The aim of this systematic review is to examine the definition, frequency, nature, and severity of adverse effects occurring parallel to or following psychotherapeutic treatment and to compare it against control groups.

**Methods:**

All registered randomised controlled trials published since 2004 (publication year of harm-reporting extension of the CONSORT statement) with adult patients fulfilling clinical criteria of defined mental disorders, which compare individual or group psychotherapy against a control group, will be included. First, a search through international trial registers as well as a search in literature databases (e.g. MEDLINE) and in relevant journals (e.g. Trials) for study protocols will be conducted to identify eligible trials. In a second step, we will search for respective publications of the results of the eligible studies. Publications will be retrieved and screened for eligibility. Two previously trained, independent raters will extract the data in duplicate. Reporting of adverse effects will be descriptively analysed regarding frequency, heterogeneity, and longitudinal course. We will further compare the adverse effects of psychotherapeutic interventions against various control groups. For each categorical outcome, we will calculate relative risks (RR) together with 95% confidence intervals. For continuous outcomes, standardised mean differences (Hedges’ *g*) with a 95% confidence interval will be computed. Between-study heterogeneity will be tested with the *Q* statistic and quantified using *I*^2^.

**Discussion:**

Preselecting studies with regard to randomised controlled trials might induce bias due to dropout before the beginning of treatment or end of treatment. However, we will thoroughly assess the negative effects of randomisation, e.g. reasons for non-randomisation, if reported. Even if delayed adverse effects might be overlooked in randomised controlled trials, these are the only sources of causal evidence.

**Systematic review registration:**

PROSPERO International Prospective Register of Systematic Reviews 2017: CRD42017055507 (17 January 2017)

## Background

It has been argued that any intervention with the potential of relieving mental distress also bears the risk of eliciting adverse effects [1]. Various attempts have been made to define adverse effects in the course of psychotherapeutic treatments, whereby the following terms have been suggested [2–4]: All adverse events occurring parallel to or following treatment are termed *unwanted event(s)*. Based on the supposed causality of the adverse events, different additional terms have been suggested, such as *adverse treatment reactions* or *side effects* (caused by correctly applied treatment) and *malpractice reactions* (caused by incorrectly or improperly applied treatment). Adverse events that require some form of high-intensity treatment are referred to as *severe adverse events*. Due to their seriousness, severe adverse events should always be addressed, irrespective of causality. If treatment does not result in positive change, patients could show either *treatment non-response* (no clinically meaningful positive change after therapy) or *deterioration of illness* (clinically meaningful negative change after treatment). Both non-response and deterioration can be causally related to the treatment or not. Regarding psychotherapy, there are a number of potential adverse effects which are discussed, ranging from worsened or novel symptoms, such as symptom substitution [4–8], to dependence from the therapist [9], stigmatisation [10], relationship problems or even separation [11, 12], as well as misuse of alcohol or drugs, deliberate self-harm, and suicidal ideation or attempts [4].

To what extent adverse effects exist and pose a problem in psychological treatments is, however, a topic of great debate with a long history in clinical research [4, 13]. Since “reporting harms may cause more trouble and discredit than the fame and glory associated with successful reporting of benefits” (p. 66) [14], such reporting has long been neglected [15, 16]. Meanwhile, adverse event monitoring is part of Good Clinical Practice and is ethically required by Institutional Review Boards, also for non-invasive interventions such as psychological interventions and, more specifically, psychotherapy. While the reporting of adverse effects in biomedical research (e.g. pharmacotherapy) usually is based on a straightforward definition of harmful events, psychological interventions still struggle with a consensus of how to define more subtle psychological adverse effects in collaborative interventional settings [17–20]. Taking an encompassing position, we will focus comprehensively on *adverse effects*, including non-response, deterioration, and adverse events, perceived as negative, uncomfortable, or harmful either by the patient, the therapist, or significant others, which were not means of therapy [21].

At present, there is a broad and somewhat inconsistent handling of how to define and report data on the rate and type of adverse effects in psychotherapy trials [3]. At least some evidence is available to estimate rates of non-response or deterioration in patients treated with psychotherapy. Meta-analyses on the efficacy of different forms of psychotherapy suggest that up to 50% of the patients do not show clinically significant change, and in about 5–20% of patients, adverse events, including treatment failure and deterioration of symptoms, emergence of new symptoms, suicidality, occupational problems or stigmatisation, changes in the social network or strains in relationships, therapy dependence, or undermining of self-efficacy, should be expected [3, 22–24].

Moreover, there are some psychological interventions that might induce harm in a significant number of patients. A review from 2007 lists some psychological interventions that are potentially harmful, such as critical incidence stress debriefing, grief counselling for normal bereavement, and boot camp interventions for conduct disorder [22]. In their review of the efficacy and harm of psychotherapy, Wampold and Imel [20] point to the necessity of randomised controlled trials (RCTs) to detect adverse effects that are causally related to the treatment. They conclude that findings of this type are relatively rare and mostly apply to preventive interventions and/or psychological interventions that would not be considered psychotherapy.

Accurate and complete safety data are indispensable for the proper evaluation of the benefit-to-harm ratio of interventions in a medical context [25]. In psychological interventions, most of the existing systematic reviews primarily focus on the efficacy or effectiveness of broad outcomes related to psychological suffering, such as reduction of psychological disorders and related symptoms, goal attainment, enhancement of well-being, and/or psychosocial functioning [26]. Basically, to make a balanced decision about any intervention, it is essential to estimate comprehensive evidence according to the benefits as well as the adverse effects [27]. Even though the magnitude of reporting adverse effects has increased over time, only 27% of the reviews on the evaluation of any type of therapeutic intervention published between 1996 and 2000 included information about safety, and only 4% explicitly reported prescriptive safety indicators of the intervention reviewed [28]. More recently, Meister et al. [17] found that merely one out of nine psychotherapy trials on persistent depressive disorder reported any information on adverse events (whereas 39 from 42 pharmacological studies published such data).

Currently, there is no systematic review focusing on the quantitative evaluation of adverse effects of psychotherapy; the International Prospective Register of Systematic Reviews (PROSPERO; https://www.crd.york.ac.uk/PROSPERO/; accessed 20 Nov 2017) does not indicate any ongoing review dealing with adverse effects of psychotherapy. There may be systematic adverse effects associated with psychotherapy. Without understanding the nature and prevalence of adverse events, patients cannot be informed adequately about the possible risks and benefits of psychotherapeutic interventions prior to engaging in treatment [15]. Moreover, it is a recent claim in many psychotherapy contexts that there is a need to find a consensus on a more systematic definition and assessment of adverse events and outcomes [4, 17–19, 21].

The proposed systematic review and meta-analysis aims to:

1. Record the definition of adverse events in primary studies;
2. Assess the extent to which adverse events are specified in psychotherapy trial protocols and publications of psychotherapy trial results in general;
3. Systematically examine the frequency, nature, and severity of adverse events occurring parallel to or in the wake of psychotherapeutic treatment;
4. Descriptively contrast the positive and negative effects of psychotherapy to guide practitioners towards a balanced treatment decision;
5. Compare the frequency, heterogeneity, and longitudinal course of adverse effects of psychotherapeutic interventions against various control groups (untreated control groups, treatment-as-usual control groups, unspecific treatment control groups, pharmacotherapy or other psychotherapy interventions, head-to-head comparison);
6. Identify relevant moderators of the frequency of adverse events (e.g. type of treatment, type of mental disorder); and
7. Examine the attribution of adverse effects within the studies (e.g. treatment vs. therapist vs. patient effects vs. other).

By focusing on a broad research question, we will be able to evaluate a variety of potential adverse effects and will provide a much more comprehensive view of the problems which appear. Our review has the potential to be used as part of a scoping exercise to identify specific adverse events that merit a further, more detailed look in future studies using a more narrowly focused approach [29].

After a period of research supporting the generally positive effects of psychotherapy, it is time to take a closer look at adverse effects, predominantly to improve the quality of psychological treatments, both in research designs and general practice [4, 17–19, 21, 27, 29]. Teaching and educating patients and therapists about these effects will have the potential to improve the outcome of existing treatments, to modify existing treatments, and to improve the choice between different treatments based upon more precise figures which reflect adverse effects. In clinical research, increased knowledge about adverse effects will stimulate the development of specific measures for these effects and encourage routine inclusion of these measures into clinical studies. Given the high prevalence of mental disorders [30–32] that are treated by psychotherapy to a large extent [33], and the great relevance of considering adverse effects in clinical decision-making [22], addressing the research issues mentioned is important for both patients and clinicians.

## Methods/design

### Design of primary studies

We will limit the inclusion to RCTs with a published trial protocol published since 2004 (year of publication of the Consolidated Standards of Reporting Trials [CONSORT] extension for harms). Randomised trials with adequate sample size offer a unique opportunity to assess the frequency and severity of adverse events in a controlled and objective setting, with the most comprehensive and systematic accumulation of pertinent information. Such information is essential to estimate benefit/harm ratios in the application of medical interventions [34, 35]. Moreover, it has been stated that the most valid estimates of non-response and deterioration effects can be obtained from comparisons of randomly assigned treatment and non-treatment groups [36].

Registered study protocols are recommended as a prerequisite for conducting clinical studies [37]. The Declaration of Helsinki states that “Every clinical trial must be registered in a publicly accessible database before recruitment of the first subject” (para 35) [38]. Furthermore, the SPIRIT 2013 Statement provides recommendations for a minimum set of scientific, ethical, and administrative elements that should be addressed in a clinical trial protocol [39]. Accordingly, a study protocol should contain plans for collecting, assessing, reporting, and managing solicited and spontaneously reported adverse events and other unintended effects of trial interventions (item 22). Key considerations should include the severity of the adverse event, determination of potential causality, and whether it represents an unexpected or anticipated event [39]. Hence, in clinical trials with a published study protocol, the probability of systematically assessed and reported adverse events might be more systematised as in studies without a published study protocol.

### Population

#### Inclusion criteria

We will include adult patients fulfilling clinical criteria of the following mental disorders based upon DSM-5: mood disorders (296, 300.4, 311, 301.13); anxiety disorders (300), including obsessive-compulsive disorders (300.3), trauma- and stressor-related disorders (308.3, 309.81); and personality disorders (301). If DSM-III/DSM-IV or ICD-9/ICD-10 is used in a trial for the diagnosis of the respective disorder, the study will be included as well. Undoubtedly, besides severe and persistent mental illness and addictions, these three diagnostic subgroups represent the epidemiologically most relevant clinical disorders within the psychotherapeutic health service system and psychotherapy research [40].

#### Exclusion criteria

Studies with children and adolescents (mean age 18 or younger) will be excluded. Although research of adverse effects of psychotherapy has focused on adults until now, there is some evidence that younger people are potentially more vulnerable which goes along with a higher prevalence of negative therapy results [16, 41]. Moreover, especially, children need specific support to express adverse effects, and children as well as adolescents may not be free to end treatment, both of which might negatively impact the informative value of the assessments of adverse events. Because of these potential concerns in younger populations, we decided to exclude these specific patient populations [16].

### Intervention(s)

Eligible trials will examine the effects of individual and group psychotherapy (as defined by the authors of the primary studies) based upon a psychotherapeutic formal change theory (cognitive behavioural, psychodynamic, interpersonal, systemic, humanistic-experiential, integrative) performed by a professional therapist. Telemedical interventions (e.g. Internet-delivered psychotherapy) will be eligible as long as all other inclusion criteria are fulfilled, i.e. there is at least some therapist contact. Interventions systematically combining psychotherapy and pharmacotherapy will be excluded due to possibly confounding intervention effects.

### Comparator(s)

Control interventions will be grouped into different categories of untreated control group (wait list), treatment-as-usual (TAU) control groups, treatment control groups (attention control/non-bona fide psychotherapy/treatment other than psychotherapy, e.g. exercise/pill placebo), pharmacotherapy or bona fide psychotherapy interventions (head-to-head comparison).

### Outcomes

According to the definitions specified above, we will include the following outcomes [2, 3]:

- Adverse event (number of participants with treatment-emergent reactions, adverse treatment reactions or side effects, malpractice reactions),
- Treatment non-response (number of participants with lack of clinically meaningful [positive] change after treatment),
- Deterioration of illness (number of participants with clinically meaningful [negative] change after treatment).

Moreover, we will include treatment response as well as primary and secondary outcomes (as defined by the authors) in order to produce a comprehensive picture of the effects and side effects of psychotherapy. Outcomes will be considered when measured post-treatment and at follow-up. However, we do not want to focus on premature termination since the evidence concerning this topic was recently summarised in systematic reviews [42–44]. The respective definitions of non-response and deterioration will be based on the criteria that the primary studies have used.

### Search strategies

Search approaches reliably yielding all the studies that have data on adverse effects of an intervention will require a comprehensive search that might be of low precision [29]. Therefore, a common literature search strategy within existing databases (“top-down” strategy) would probably lead to an unmanageable amount of records to be screened for eligibility. Such a search strategy is likely to contain most of the relevant studies, but if only a small proportion is relevant, it is very imprecise and resource intensive [29]. To identify relevant RCTs more efficiently, we will use a “bottom-up” strategy. In the first step, we will look for eligible protocols of primary studies, and in the second step, we will look for respective publications of results. Therefore, common international trial registers (e.g. clinicaltrials.gov) will be screened for eligible studies. Additionally, a comprehensive search will be performed in databases (MEDLINE, CENTRAL, PsycInfo, Web of Science) for study protocols of randomised controlled psychotherapy trials by using a combination of relevant keywords (psychotherapy AND (rct OR rando*) AND protocol), limited by date of publication (01 January 2004 to present). Moreover, we will conduct similar searches within the databases of relevant journals (i.e. BMC Psychiatry, BMC Psychology, BMJ Open, Contemporary Clinical Trials, Trials) without “protocol” as a keyword. Titles and abstracts of identified study protocols will be independently screened by two reviewers. Full reports of all protocols that appear to be eligible or left any uncertainty will be obtained and checked for inclusion. Disagreements will be resolved through discussion. In case of excluding a trial, reasons will be documented. Subsequently, we will search for respective publications of eligible studies by searching in databases (i.e. MEDLINE, CENTRAL, PsycInfo, Web of Science) via the title, author names, and, if possible, the trial registration number and contacting the authors of relevant study protocols. After being retrieved, the studies will be screened once again for eligibility by two reviewers as described above. Records and full texts will be managed by using Endnote X.

### Data extraction

First, a coding form and an additional coding manual will be developed. Then, the coding procedure will be tested in a pilot phase, followed by potential modifications of the form. The coding form will allow for the standardised description and evaluation of substantial and methodological features possibly influencing the effect sizes of the integrated studies. To assure high reliability of the coding, two previously trained, independent raters will extract the data in duplicate. Disagreement will be resolved via consensus discussion or consultation with a third person (i.e. principal investigator).

The comparison between the psychotherapeutic intervention and the control group will be defined as a unit of analysis (comparison level). We will extract the following data:

- ▪ General information about the research report (e.g. authors, title, publication year, publication format, source of funding, publication language, country of performance);
- ▪ Characteristics of the patients (e.g. age, gender, partnership, socioeconomic status, primary diagnosis, comorbidity, additional treatments);
- ▪ Characteristics of intervention and therapists (e.g. type and theoretical background of treatment, length and frequency of the intervention, number of therapists providing treatment);
- ▪ Characteristics of the control group (e.g. type of control group, level of standardisation);
- ▪ Methodological features (e.g. inclusion and exclusion criteria; recruitment-, participation- and dropout rates for each group and reasons for dropout; prospectively planned analyses of therapist effects; therapist effects considered in the data analysis of outcomes and negative effects);
- ▪ Characteristics of outcome variables:
  - Type of outcome/adverse event;
  - Characteristics of an adverse event: serious, non-serious, intervention-related or independent of the intervention, side effect (related to the patient or his/her social environment), for non-response/deterioration: type of assessment;
  - Quality of information on adverse effects: exhaustiveness, accordance of the study protocol and report of results (i.e. pre-specified in protocol vs. not pre-specified).
  - Surveillance of adverse event: active vs. passive, anticipated vs. unanticipated, solicited vs. unsolicited;
  - Time point of assessment/occurrence (post, follow-up; phase of psychotherapy);
- ▪ Effect size related parameters (frequency of adverse events, non-response, and deterioration; sample size)

Risk of bias will be assessed by using the Risk of Bias tool RoB 2.0 of the Cochrane Collaboration [45]. Risk of bias assessment will be conducted by two independent raters, and disagreement will be resolved via consensus discussion or consultation of a third person.

### Data synthesis

First, we will descriptively assess the extent to which adverse events are specified in psychotherapy trial protocols and publications of psychotherapy trial results in general. Second, the frequency, nature, and severity of adverse events occurring parallel to or in the wake of psychotherapeutic treatment will be examined. This allows us to address new adverse effects that were previously unrecognised. Adverse effects will be classified as (1) adverse event, (2) serious adverse event, (3) non-response, and (4) deterioration. We will also provide subgroup data for different mental disorders, treatment modalities, psychotherapy orientations, and treatment settings. Third, we will descriptively compare the positive and negative effects of treatment. Therefore, we will calculate standardised mean differences (Hedges’ *g*) as between-study effect sizes with 95% confidence interval pooled across all outcomes defined as primary and secondary by the authors using a random effects model (DerSimonian and Laird). In the fourth step, adverse effects of psychotherapeutic interventions will be compared against each control group category (as defined above) separately. For continuous outcomes, we will calculate standardised mean differences (Hedges’ *g*) with a 95% confidence interval. For categorical outcomes, we will calculate relative risks (RR) together with 95% confidence intervals by dividing the risk of an event (e.g. adverse event, non-response) in the intervention group by the risk of a respective event in the control group. Since zero cells (e.g. no adverse events in one group) cause problems with computation of estimates and standard errors, we will add 0.5 to each cell of the 2 × 2 table for any such study. We will calculate pooled estimates using both fixed effects model (Mantel-Haenszel) and random effects model (DerSimonian and Laird).

Sensitivity analyses will be run for studies with adequate sample size only, excluding trials with less than 100 participants per group [46]. Additionally, we will run sensitivity analyses calculating risk differences with 95% confidence intervals as a measure of absolute effect by subtracting the risk of adverse events in the control group from the respective risk in the intervention group.

In order to identify differential effects, subgroup analyses will be conducted. A priori, we specify the type of treatment and kind of mental disorder as potential moderators. Additionally, we will conduct exploratory moderator analyses depending on the availability of data.

For analysing non-response and deterioration, the Reliable Change Index [47] will be used as the most commonly reported index of change. Analyses of non-response will be conducted using the intention-to-treat approach according to the following principle: When data on dropouts are carried forward and included in the evaluation using the last observation carried forward method, they will be included as such. When dropouts were excluded from any assessment in the primary studies (e.g. those who never returned for assessment after randomisation), they were considered to be non-responders in both intervention and control groups. Between-study heterogeneity will be tested with the *Q* statistic and quantified using *I*^2^ [48]. Results of primary study effects and summary effects will be presented in forest plots. Publication bias will be visually assessed in funnel plots and statistically analysed using Egger’s regression test [49]. Finally, we will evaluate the strength of the body of evidence using the GRADE system including considerations of within-study risk of bias, directness of evidence, heterogeneity, precision of effect estimates, and risk of publication bias [50].

## Discussion

### Limitations

One of the first groundbreaking findings regarding adverse effects of psychotherapy was that some therapists produced constantly negative effects while the therapies delivered by other therapists resulted in positive effects [51, 52]. If adverse effects are reported, it might not be clear how these are attributed. Although evidence from RCTs provides the strongest conclusions [20], uncertainty concerning the causality of adverse effects might remain; are they truly treatment effects, or actually therapist effects, or caused by other factors such as changes in the life of the patients? Hence, it is not likely that we will be able to answer these important causality questions by means of the present meta-analysis although we will at least assess if studies consider possible therapist effects in the data analyses of outcomes and negative effects. Even though causality considerations are important, it has been argued that at the recent stage of research, “the question of causality lacks pragmatic salience” (p. 23) [6]. Due to unresolved methodological difficulties, it seems reasonable to keep causality in mind while focusing on our review primarily on outcome prognosis as both a realistic and relevant goal [6].

It has been shown that the frequency of adverse events depends on the methods used for assessment. This may cause heterogeneity in the comparison between studies [53–55]. In order to face this problem, we will develop a checklist and ask the authors from eligible primary studies about any patient reports on adverse events that were not mentioned in their publications. As we will code treatment non-response and deterioration as defined by the authors of the primary studies, these definitions might differ from each other. Implementing the Reliable Change Index as a common measure will help minimising these possible variations. This meta-analysis is facing the same problems as any of its kind; difficulties performing all planned analysis may arise dependent on the number of included comparisons and adequate power.

We are aware that the preselection of studies with regard to RCTs might induce bias due to dropout before the beginning of treatment or end of treatment. However, we will thoroughly assess the negative effects of randomisation as well, e.g. reasons for non-randomisation, if reported. Because of few participants and short duration of trials, adverse events which occur seldom or delayed can be overlooked in RCTs. On the other hand, non-randomised studies bear other disadvantages, e.g. being more vulnerable to biases [29] and underestimating the absolute risk of harm [56]. Since “randomized controlled trials are the only possibility to obtain causal clinical evidence regarding AEs [adverse events] and SAEs [serious adverse events] and therefore constitute a unique and important source for patient safety information” (p. 106) [17], the advantages outweigh the disadvantages for our purpose.

## Abbreviations

RCT: Randomised controlled trial
RR: Relative risk
